# Vacuum Inner Spacer to Improve Annealing Effect during Electro-Thermal Annealing of Nanosheet FETs

**DOI:** 10.3390/mi13070987

**Published:** 2022-06-24

**Authors:** Dong-Hyun Wang, Khwang-Sun Lee, Jun-Young Park

**Affiliations:** School of Electronics Engineering, Chungbuk National University, Chungdae-ro 1, Cheongju 28644, Korea; dh.wang@chungbuk.ac.kr (D.-H.W.); ksunlee@chungbuk.ac.kr (K.-S.L.)

**Keywords:** electro-thermal annealing, nanosheet FET, reliability, vacuum inner spacer

## Abstract

Electro-thermal annealing (ETA) in a MOSFET utilizes Joule heating. The high-temperature heat effectively cures gate dielectric damages induced by electrical stresses or ionizing radiation. However, even though ETA can be used to improve the reliability of logic and memory devices, applying ETA in state-of-the-art field-effect transistors (FETs) such as nanosheet FETs (NS FETs) has not yet been demonstrated. This paper addresses the heat distribution characteristic of an NS FET considering the application of ETA, using 3D simulations. A vacuum inner spacer is newly proposed to improve annealing effects during ETA. In addition, evaluations of the device scaling and annealing effect were performed with respect to gate length, nanosheet-to-nanosheet vertical space, and inner spacer thickness. Guidelines for ETA in NS FETs can be provided on the basis of the results.

## 1. Introduction

Semiconductor devices have been shrunk beyond Moore’s law, to increase output performance and to reduce power consumption [1]. However, as chip sizes become smaller, it is becoming more difficult to both control short-channel effects (SCEs) and improve device reliability. SCEs lead to increased standby power consumption by increasing off-state current (I_OFF_) [2]. SCEs have been suppressed by the evolution of device structures such as FinFETs [3] and gate-all-around (GAA) FETs [4]. It is also possible to improve immunity to SCEs with the aid of advanced material engineering and process technology [5].

In contrast to work on SCEs, there have been few breakthrough advances to address the degradation in reliability resulting from gate dielectric aging, except for lightly doped drain (LDD) and forming gas annealing (FGA), which were developed decades ago [6,7].

Recently, an electro-thermal annealing (ETA) configuration was introduced which utilizes Joule heat generated by the device itself to cure damaged gate dielectrics [8]. The ETA effectively cures the gate dielectric damage produced by hot-carrier injection (HCI), bias-temperature instability (BTI), and even total ionizing dose (TID). In addition, the ETA is applicable for removing contaminants such as photoresist (PR), moisture, or traps existing in channels [9,10]. However, additional power consumption is inevitably required to trigger the ETA.

Several approaches have been proposed to improve the annealing effect and to minimize the power consumption by modifying the device structure or materials [11,12]. However, these previous methods are not fully compatible with state-of-the-art logic transistors such as nanosheet FETs (NS FETs), since the backbone of the NS FET is completely different compared to conventional FinFETs and GAA FETs.

This paper demonstrates heat distribution characteristics during ETA in an NS FET. In addition, a novel device structure is provided which enables an improved annealing effect under identical power consumption. The proposed device structure contains a vacuum inner spacer which includes a vacuum dielectric. Device scaling was investigated in terms of gate module, multiple NS channels, and vacuum inner spacers, and the results are discussed for better use of ETA.

## 2. Device Structure and Simulation Methodology

In this study, 3D simulations of the NS FETs with a vacuum inner spacer were performed using COMSOL Multiphysics software. The reason for using COMSOL is because it is the most useful tool for analyzing heat during ETA in nanoscale devices [8,12]. Heat transfer in both the solids module and the electric currents module was used. The simulated environmental conditions were assumed to be air, and the heat transfer coefficient (*h*) for convective cooling was assumed to be 10 W/m^2^K. The mesh sizes were varied from 1 nm to 10 nm according to the structure. All simulations were performed under steady-state conditions.

**Figure 1:**
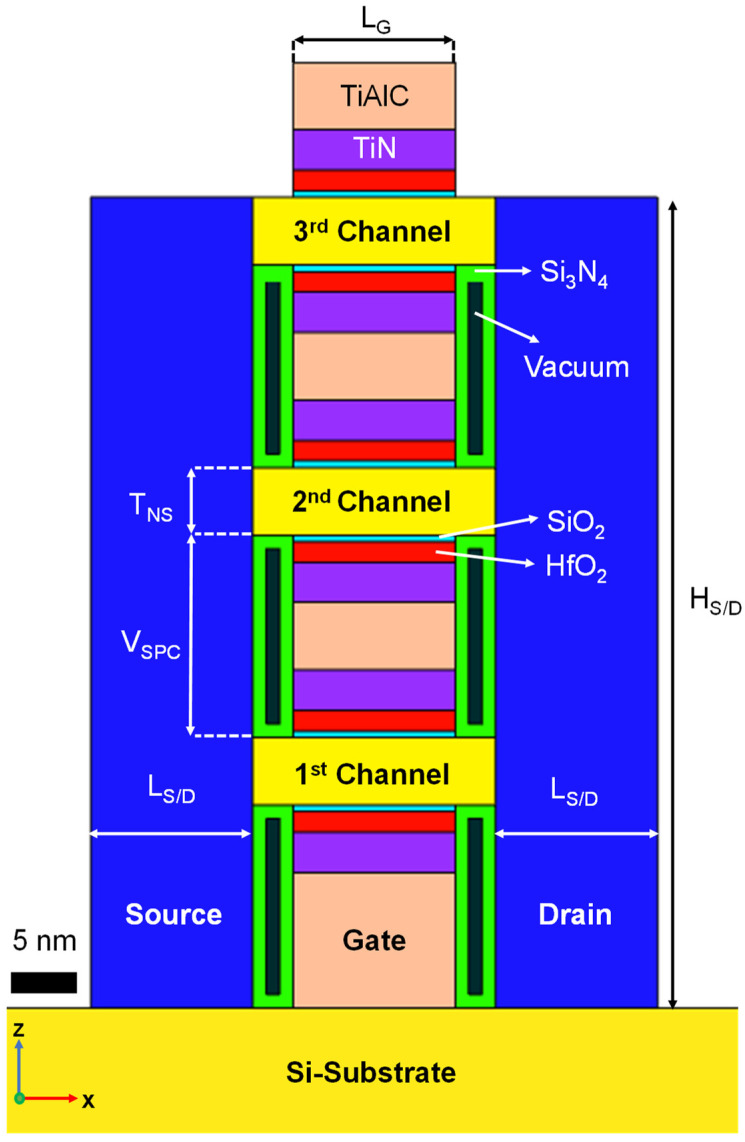
Cross-sectional schematic of an NS FET with vacuum inner spacer for ETA simulations.

Figure 1 shows a schematic of an NS FET including the vacuum inner spacer. The NS FET has three suspended channels composed of silicon which are surrounded by a high-*k* and metal gate.

The gate length (L_G_), channel width (W_NS_), nanosheet thickness (T_NS_), and nanosheet-to-nanosheet vertical space (V_SPC_) were 12 nm, 30 nm, 5 nm, and 15 nm, respectively [13]. The SiO_2_ and HfO_2_ interlayers of 1 nm and 3 nm, respectively, were deposited on suspended nanosheets as the gate dielectric. The equivalent oxide thickness (EOT) of the gate dielectric was assumed to be 1.53 nm. In terms of gate module, TiAlC was deposited on TiN as the gate metal [14]. Using vacuum spacer structures has several advantages for logic transistors [15,16,17,18,19]. In particular, in an NS FET, the vacuum inner spacer shows superior AC performance for the device with a low-*k* inner spacer. The vacuum inner spacer for this work was surrounded by a 1 nm Si_3_N_4_ outer shell. Detailed information about the device structure and materials is summarized in Table 1 and Table 2**.** Then, a current of 2.5 mA was applied through the gate-to-gate method to trigger ETA for Joule heat generation higher temperature than 600 °C [8].

## 3. Results

**Figure 2:**
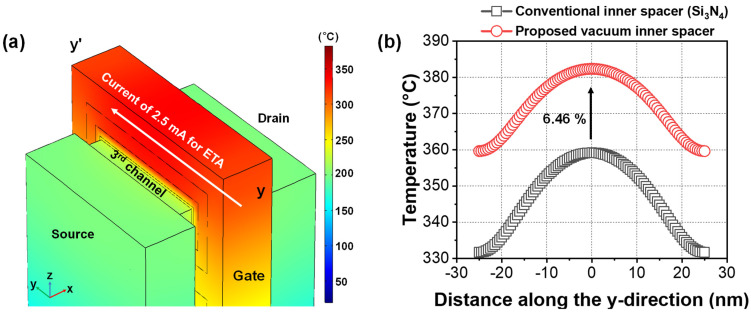
(**a**) Simulated heat distribution profile during ETA using gate-to-gate current in an NS FET. (**b**) Extracted temperature on the gate surface according to inner spacer material.

Figure 2a shows the heat distribution profile during ETA using gate-to-gate current in an NS FET. During the ETA, the gate temperature was increased beyond 300 °C by Joule heating. The temperature was highest on the surface of the gate electrode, but lowest near the Si substrate, since the Si substrate acted as a heat sink. It was observed that the temperature of the gate surface rose higher than 300 °C, while the temperature in the source and the drain regions remained relatively low because of heat dissipation through the source and drain, which had high thermal conductivity.

Figure 2b shows the extracted temperature of an NS FET with respect to the inner spacer materials. The temperature was extracted after reaching a steady state. During the ETA using gate-to-gate current, most of the Joule heat was concentrated in the middle of the gate electrode, as expected. While curing of the gate dielectric characteristics, on the basis of measured I_D_–V_G_ or gate leakage (I_G_), is not included in this work, these electrical results have already been reported several times [8].

When the vacuum inner spacer was included, the temperature during the ETA became 6.46% higher than the case with a conventional inner spacer composed solely of Si_3_N_4_. Since the thermal conductivity of the inserted vacuum was 0.024 W/(mK), the low thermally conductive vacuum provided thermal isolation during the ETA. As a result, the annealing effect induced by the ETA could be further improved but with identical power consumption. To improve the annealing effect further, it is desirable to provide guidelines related to device scaling such as L_G_, T_IN_, and V_SPC_. In particular, L_G_ is one of the aggressive design parameters in the minimization of semiconductor devices.

**Figure 3:**
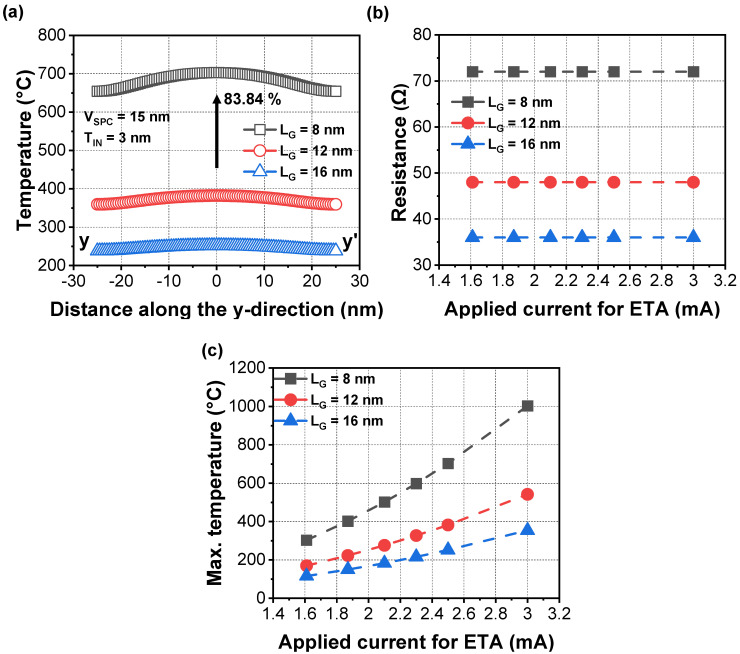
(**a**) Extracted temperature during the ETA in an NS FET with vacuum dielectric with respect to L_G_. Extracted (**b**) gate to-gate resistance and (**c**) maximum temperature with various applied currents for the ETA, respectively. Inner spacer thickness (T_IN_) was fixed to 3 nm regardless of L_G_.

Figure 3a shows the simulated gate surface temperatures considering L_G_ scaling from 16 nm to 8 nm. As the L_G_ decreased from 12 nm to 8 nm, the temperature dramatically increased over 700 °C under an identical power consumption of 2.5 mA. Temperature sensitivity was approximately 80 °C/nm of L_G_. As L_G_ decreased, the temperature increased due to the increased gate-to-gate resistance, as shown in Figure 3b. As the cross-sectional area of the gate electrode was reduced by L_G_ scaling, the gate-to-gate resistance increased. Hence, the increased voltage drop across the gate-to-gate interval raised the Joule heat temperature [25]. In addition, when current for the ETA was increased from 2.5 mA to 3 mA, the annealing temperature also increased. However, for a relatively long channel device (i.e., L_G_ = 16 nm), it was difficult to generate a sufficiently high temperature to enable gate dielectric curing, even under a current of 3 mA.

**Figure 4:**
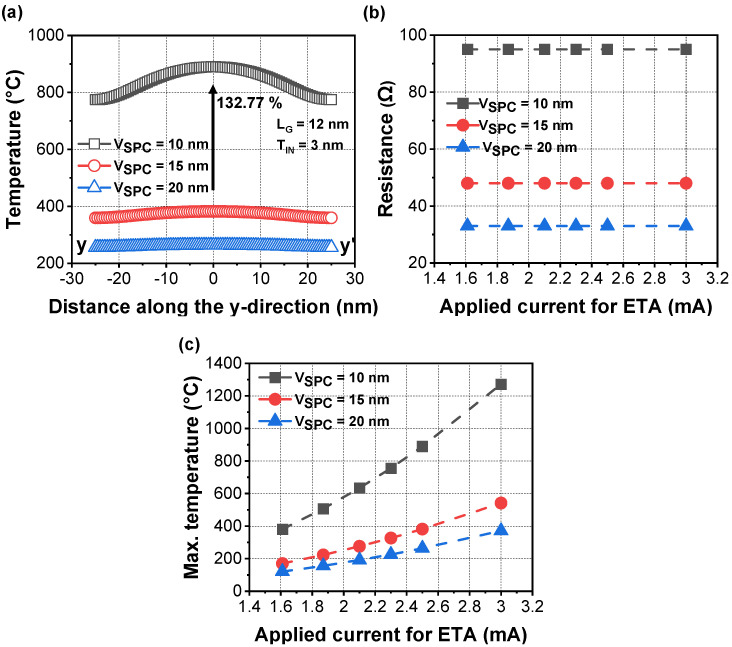
(**a**) Extracted temperature during the ETA in an NS FET with vacuum dielectric with various V_SPC_. Extracted (**b**) gate-to-gate resistance and (**c**) surface temperature with respect to V_SPC_ and applied current, respectively.

Figure 4a shows the simulated surface temperature when V_SPC_ was reduced from 20 nm to 10 nm. The temperature during the ETA increased as the V_SPC_ decreased. The temperature sensitivity with respect to V_SPC_ was approximately 101 °C/nm. Considering the extracted sensitivities, the impact of V_SPC_ was greater than the case with L_G_ scaling. When the V_SPC_ decreased, metal gate height also decreased; hence, the gate-to-gate resistance became larger, as shown in Figure 4b. The increased gate-to-gate resistance gave rise to higher generated Joule heat temperature, as described above. Typically, the Joule heat temperature depends on the applied current density, as shown in Figure 4c. However, when V_SPC_ was longer than 20 nm, insufficient Joule heat was generated to be used for the ETA because of the lowered gate-to-gate resistance. Hence, a V_SPC_ narrower than 15 nm would be preferred for a better annealing effect of NS FETs. This tendency coincides with the development trend of current NS FETs toward a narrower V_SPC_ for better packing density [26].

**Figure 5:**
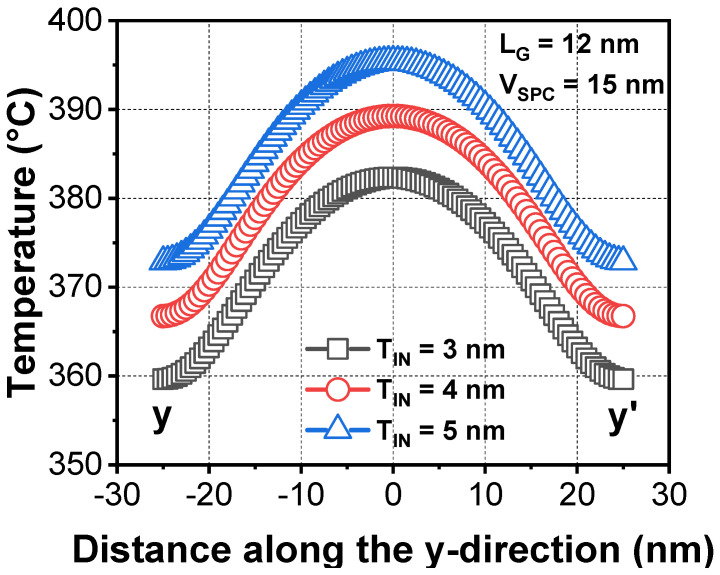
Extracted temperature during the ETA with various inner spacer thickness (T_IN_).

Figure 5 shows the simulated surface temperature according to vacuum inner spacer thickness (T_IN_). Modification of the T_IN_ was independent of the gate-to-gate resistance (not shown). However, the temperature sensitivity during the ETA decreased by approximately 6 °C/nm, as the T_IN_ became thinner. This was the effect of increased heat dissipation. As the T_IN_ decreased, thermal resistance in the source/drain (S/D) extension decreased because of reduced extension length. Hence, heat loss through the S/D extension increased [27].

**Figure 6:**
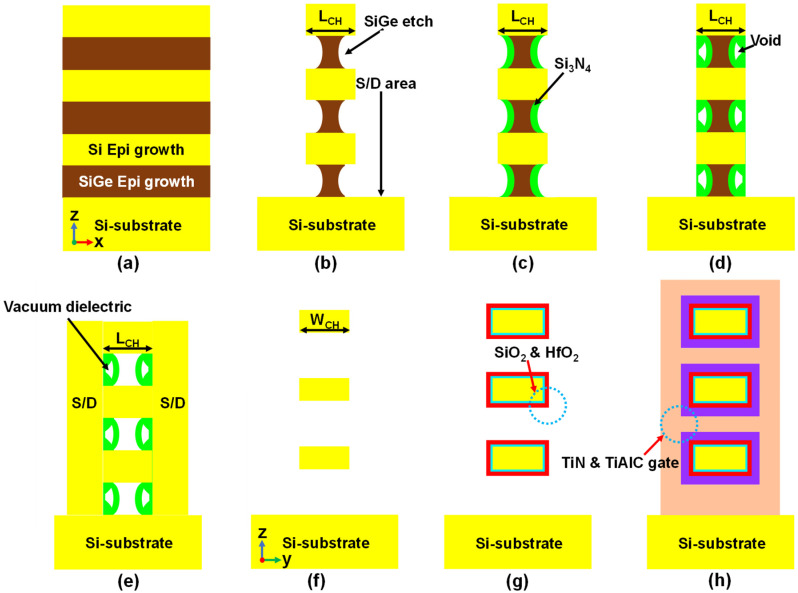
Proposed fabrication process flow of an NS FET with vacuum inner spacer. (**a**) Si/Si_x_Ge_1−x_ epitaxial growths on Si substrate. (**b**) Si_x_Ge_1−x_ indent etching. (**c**,**d**) Inner spacer deposition with void formation. (**e**,**f**) S/D epitaxial growth and suspended multiple nanosheet formation. (**g**,**h**) Gate dielectric and metal gate deposition.

Figure 6 shows the proposed fabrication process flow for an NS FET with a vacuum inner spacer. Multiple Si/Si_x_Ge_1−x_ layers are deposited on the Si substrate using iterative epitaxial growth. After dry etching to form the S/D region, the Si_x_Ge_1−x_ is selectively indented etched, as shown in Figure 6b. Then, Si_3_N_4_ for the shell of the vacuum dielectric is deposited using a poor step coverage process such as plasma-enhanced chemical vapor deposition (PECVD), as shown in Figure 6c,d. The void acts as a vacuum dielectric. Thereafter, nanosheets are released and suspended during the replacement of the poly-Si gate (RPG) process in Figure 6e. Finally, gate stacks including the gate dielectric and work function adjustment metals are deposited.

## 4. Conclusions

The heat distribution characteristics during the ETA of an NS FET were demonstrated using 3D simulations. When the inner spacer in an NS FET was replaced by a vacuum inner spacer instead of the conventional Si_3_N_4_ inner spacer, the temperature generated during the ETA increased by 6.46% under identical power consumption. Device scaling with respect to L_G,_ V_SPC_, and T_IN_ was investigated in relation to the ETA temperature. The V_SPC_ scaling showed a significant temperature increase with a sensitivity of 101 °C/nm. Moreover, L_G_ scaling showed a temperature increase of 80 °C/nm. However, T_IN_ scaling resulted in a decrease in ETA temperature, and the sensitivity was negligible. As a result, it can be concluded that the proposed NS FET with a vacuum inner spacer is a promising candidate to improve annealing during ETA.

## Figures and Tables

**Table 1:** Device geometric information for simulations.

Parameters	Materials	Values
Gate length, L_G_	TiAlC	12 nm [20]
Channel width, W_NS_	Si	30 nm [13]
Nanosheet thickness, T_NS_	Si	5 nm [13]
Inner spacer thickness, T_IN_	Si_3_N_4_	3 nm [13]
Nanosheet-to-nanosheet vertical space, V_SPC_	-	15 nm
Gate dielectric	SiO_2_/HfO_2_	1/3 nm
Source/drain length, L_S/D_	Si	12 nm [13]
Source/drain height, H_S/D_	Si	60 nm
Vacuum dielectric	Vacuum	1 nm

**Table 2:** Material properties for simulations.

Material	Relative Permittivity	Thermal Conductivity (W/mK)	Electrical Conductivity (S/m)
Si	11.90 [21]	Si substrate 140 [22]	7.68 × 10^−3^
		Channels 18 [22]	7.68 × 10^−3^
		Source/drain 38 [22]	5 × 10^5^
Si_3_N_4_	7.50 [23]	3.2	1 × 10^−8^
SiO_2_	3.90 [21]	1.4	1 × 10^−17^
HfO_2_	22	1.06	1 × 10^−14^
TiAlC	1	46	2. × 10^6^
Vacuum	1 [21,23]	0.024 [24]	1 × 10^−15^
